# Prevalence of intestinal parasites and molecular characterization of *Giardia intestinalis*, *Blastocystis* spp. and *Entamoeba histolytica* in the village of Fortín Mbororé (Puerto Iguazú, Misiones, Argentina)

**DOI:** 10.1186/s13071-021-04968-z

**Published:** 2021-10-01

**Authors:** Ernesto Candela, Carolina Goizueta, M. Victoria Periago, Carla Muñoz-Antoli

**Affiliations:** 1 Parasitology Section, Department of Pharmacy Pharmaceutical Technology and Parasitology, School of Pharmacy, University of Valencia, Burjassot-Valencia, Spain; 2 Mundo Sano Foundation, Buenos Aires, Argentina; 3 National Scientific and Technical Research Council (CONICET), Buenos Aires, Argentina

**Keywords:** Soil-transmitted helminths, Intestinal parasites, Molecular characterization, *Giardia intestinalis*, *Blastocystis* spp., *Entamoeba histolytica*, Puerto Iguazú, Misiones, Argentina

## Abstract

**Background:**

Intestinal parasites (IPs) are widely distributed worldwide and are one of the major contributors to gastrointestinal disease. Their prevalence is associated with poor access to water, sanitation and hygiene (WASH). The objective of this study was to identify the prevalence of IPs, including soil-transmitted helminths (STH), and their relation to socioeconomic characteristics, as well as a first approach to molecularly characterize the types of *Giardia intestinalis*, *Blastocystis* spp. and *Entamoeba histolytica* present in an indigenous community from Puerto Iguazú, Misiones, Argentina.

**Methods:**

A cross-sectional study was conducted in the rural settlement of Fortin Mbororé between January and March 2018. Socioeconomic variables, household characteristics, and stool and blood samples were collected. Standard coprological techniques were used to analyze stool samples, and a complete hemogram was performed on the blood samples. *Giardia intestinalis* microscopy-positive samples were genetically typed by the β-giardin (*bg*) gene. Molecular identification of *Blastocystis* spp. subtypes and *E. histolytica* were carried out by amplification and sequencing of a partial fragment of the small subunit ribosomal RNA gene (SSU rDNA).

**Results:**

The overall prevalence of IPs was 92.7%, with 72.0% specifically for hookworm. IPs were significantly more prevalent in preschool- and school-age children (*P* < 0.05). No formal education (*P* = 0.035), the presence of unimproved floors (*P* = 0.001) and overcrowding (*P* = 0.005) were significantly associated with IP infection. Hookworm was associated with anemia (*P* = 0.019). Molecular characterization revealed the presence of *E. histolytica* sub-assemblages AII (12.5%), AIII (87.5%) and BIV (100%); one case of sub-assemblage D for *G. intestinalis*; and the presence of subtypes ST1 (14.8%), ST2 (14.8%) and ST3 (70.4%) of *Blastocystis* spp.

**Conclusions:**

Protozoans detected in this study are transmitted mainly through water contaminated with fecal matter, evidencing the need to improve the quality of water and sanitation for the inhabitants of Fortín Mbororé. Molecular characterization showed that domestic animals can be implicated in the zoonotic transmission of *G. intestinalis* and *Blastocystis* spp. to humans. A hyperendemic area for STH was found, with hookworm prevalence greater than 50%. Therefore, improvements in WASH as well as mass deworming programs need to be implemented in this area to control and decrease the prevalence of IPs in general and STH in particular.

**Graphical Abstract:**

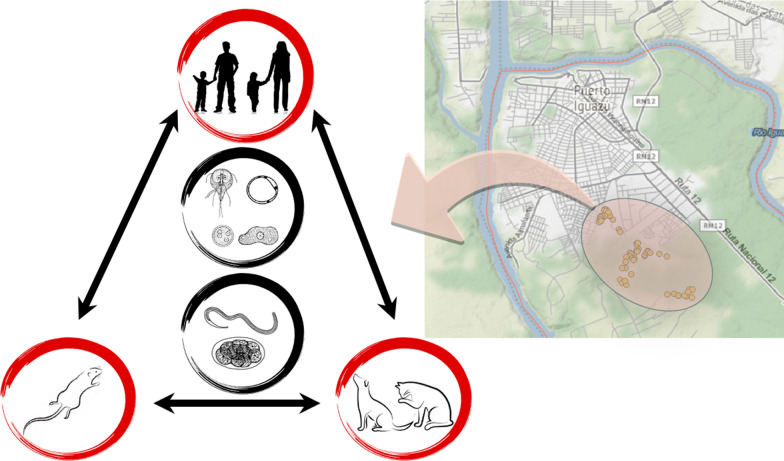

**Supplementary Information:**

The online version contains supplementary material available at 10.1186/s13071-021-04968-z.

## Background

Intestinal parasite infections (IPIs) are a global public health problem due to their high prevalence and worldwide distribution, especially in populations from tropical and subtropical areas of the developing world [1]. Although they can affect any age group, children are the most affected by the consequences of infection [1, 2]. Some of the most important causal agents are protozoans (e.g. *Entamoeba histolytica*, *Giardia intestinalis*) and helminths, with soil-transmitted helminths (STH, referring to *Strongyloides stercoralis, Ascaris lumbricoides, Trichuris trichiura* and hookworm) the most prevalent [2], and listed as part of the neglected tropical diseases (NTDs) by the World Health Organization (WHO) [3]. Several studies reported varying prevalence of IPIs in the population, which depends on socioeconomic status, sanitary and environmental conditions and access to water, as well as changes in lifestyle as a result of acculturation and environmental degradation processes [4–7].

Even though most of these infections are asymptomatic, intestinal parasites (IPs) cause malabsorption, nutritional syndromes, malnutrition, morbidity and deficiencies in children’s growth [8, 9]. Moreover, STH infections are implicated in the etiology of iron-deficiency anemia (IDA) in developing countries [10]. Specifically, moderate and heavy hookworm infections have been strongly associated with the development of anemia, due to chronic intestinal blood loss [11], as well as with cognitive impairment in school-age children and negative impact on psychomotor and language development of preschool-age children [12, 13]. All these factors contribute to the economic impact of the disease and perpetuation of poverty, causing more than 100,000 annual deaths [2] and susceptibility to develop other diseases as adults [14].

Several studies have described the presence of IPs in northern Argentina, showing a high prevalence of protozoans with a predominant presence of *G. intestinalis* and *Blastocystis* spp., as well as STH [5, 15]. Additionally, Argentina has a heterogeneous prevalence of STH infections, with high prevalence in the north [16]. Anemia is considered a public health problem in the country, particularly in the northwest, where 38% of preschool-age children and 19% of women are anemic [17]. However, the prevalence found could be higher than the one reported due to difficulty in diagnosis if the parasite load is low and given that larval/egg output tends to be irregular in helminths [18]. Moreover, the diagnostic methods commonly used for STH detection have a low sensitivity for *S. stercoralis* or fail to detect it altogether [19, 20].

Resolution WHA62.12 from the WHO World Health Assembly (WHA) urges authorities to “implement interventions against neglected tropical diseases” [21] for STH in endemic areas based on mass drug administration (MDA), normally using a single dose of either albendazole or mebendazole for *A. lumbricoides*, *T. trichiura* and hookworm, and ivermectin for *S. stercoralis*. Currently, Argentina does not have a deworming program at either the national or provincial level.

Although several epidemiological studies have been carried out in northern Argentina, none of them have delved into the molecular aspect of *G. intestinalis*, *Blastocystis* spp. and *E. histolytica*. These enteric protozoans, including *Cryptosporidium* spp., are regarded as the most common and important causes of protozoan-diarrheal disease in humans globally. Even though the effects caused by *Blastocystis* spp. are still debatable, several pieces of evidence have emerged pointing to its pathogenic role in intestinal disorders [22, 23]. Determining the molecular frequency and subtype of these protozoans is important to ascertain the sources of infection, transmission dynamics and zoonotic potential [23].

*Giardia intestinalis*, one of the major worldwide contributors to diarrheal disease, is a complex formed of eight genotypes identified to date (A–H), with several sub-assemblages; but only genotype A and B have been associated with human infections [24]. With respect to *Blastocystis* spp., based on its high level of genetic diversity, it is classified into different global ribosomal subtypes (STs). Currently, 17 subtypes have been described in different areas, with ST1 to ST9 and ST12 colonizing humans. In humans from Europe, ST1, 2, 3 and 4 reportedly occur most commonly, whereas ST1, 2 and 3 commonly occur in South America [22, 25].

The aim of this study was to identify the prevalence of intestinal parasites, including STH, due to the presence of living conditions appropriate for their transmission; identify any association between prevalence and socioeconomic characteristics; and perform a first approach to molecularly type *G. intestinalis*, *Blastocystis* spp. and *E. histolytica* present in an indigenous community from Puerto Iguazú, Misiones, Argentina. The results from this study constitute the first published report on *G. intestinalis* and *Blastocystis* genotypes circulating in the province of Misiones.

## Methods

### Study area and study population

**Fig. 1:**
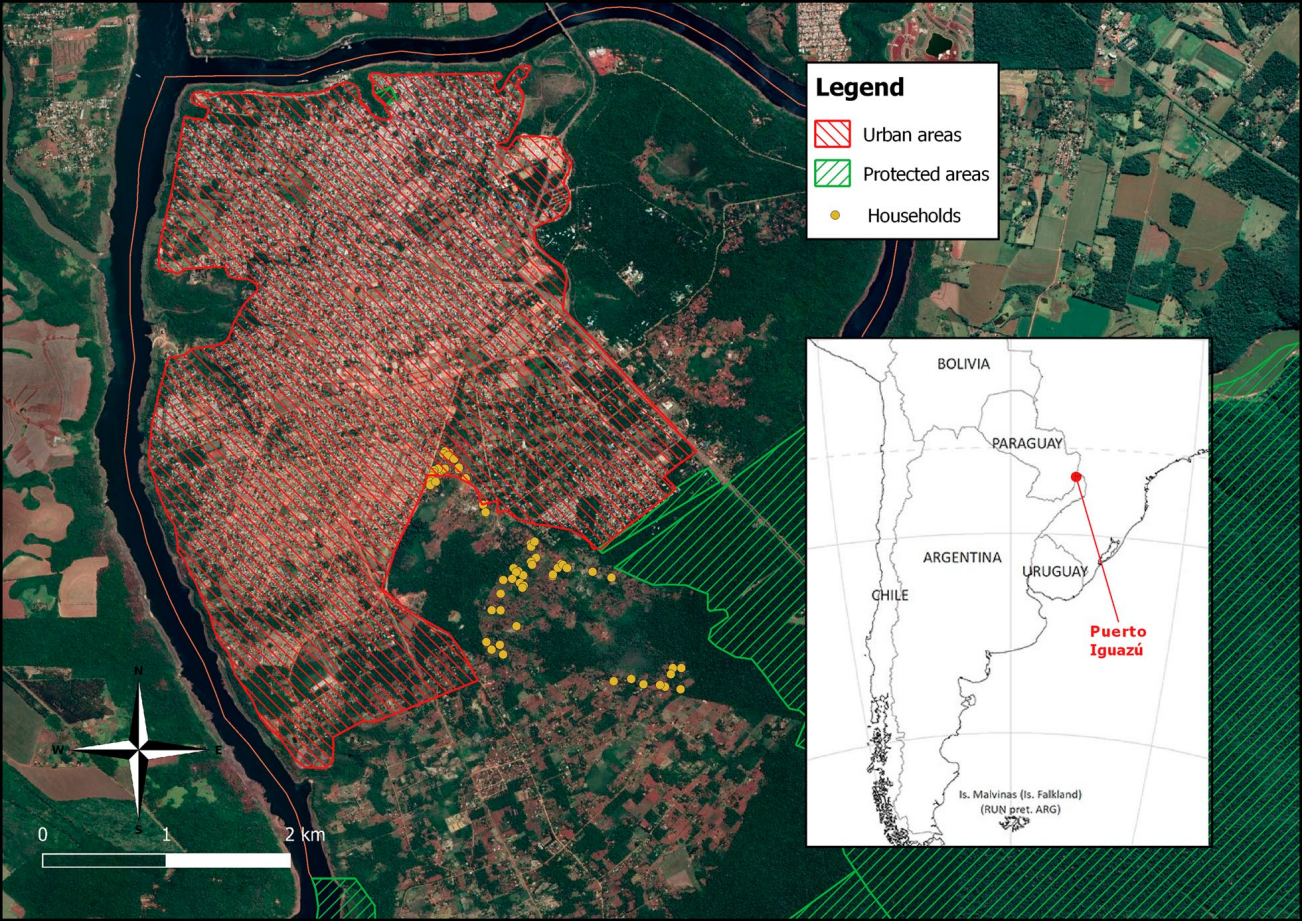
Map of the study area. The households from the village of Fortín Mbororé are shown as yellow dots, which are located just outside the urban area of the city of Puerto Iguazú, Misiones, Argentina. South America image was obtained from www.mapasparacolorear.com. This map was created with QGIS 3.14 (www.qgis.org)

This study was conducted in the city of Puerto Iguazú, located in the province of Misiones (25° 35′ 52″ S, 54° 34′ 55″ W), a subtropical province of northeastern Argentina (Fig. 1) and part of the most biodiverse region of the country [26], which houses the Iguazú National Park and Iguazú Falls. Puerto Iguazú is in the tri-border area of Argentina, Brazil and Paraguay; the three countries are naturally divided by the Paraná and Iguazú Rivers. The region is characterized by a subtropical climate with no dry season. The median annual temperature is 21 °C, with annual rainfall of 1883.2 mm [27]. The predominant soil type is lateritic of deep red color [26].

**Fig. 2:**
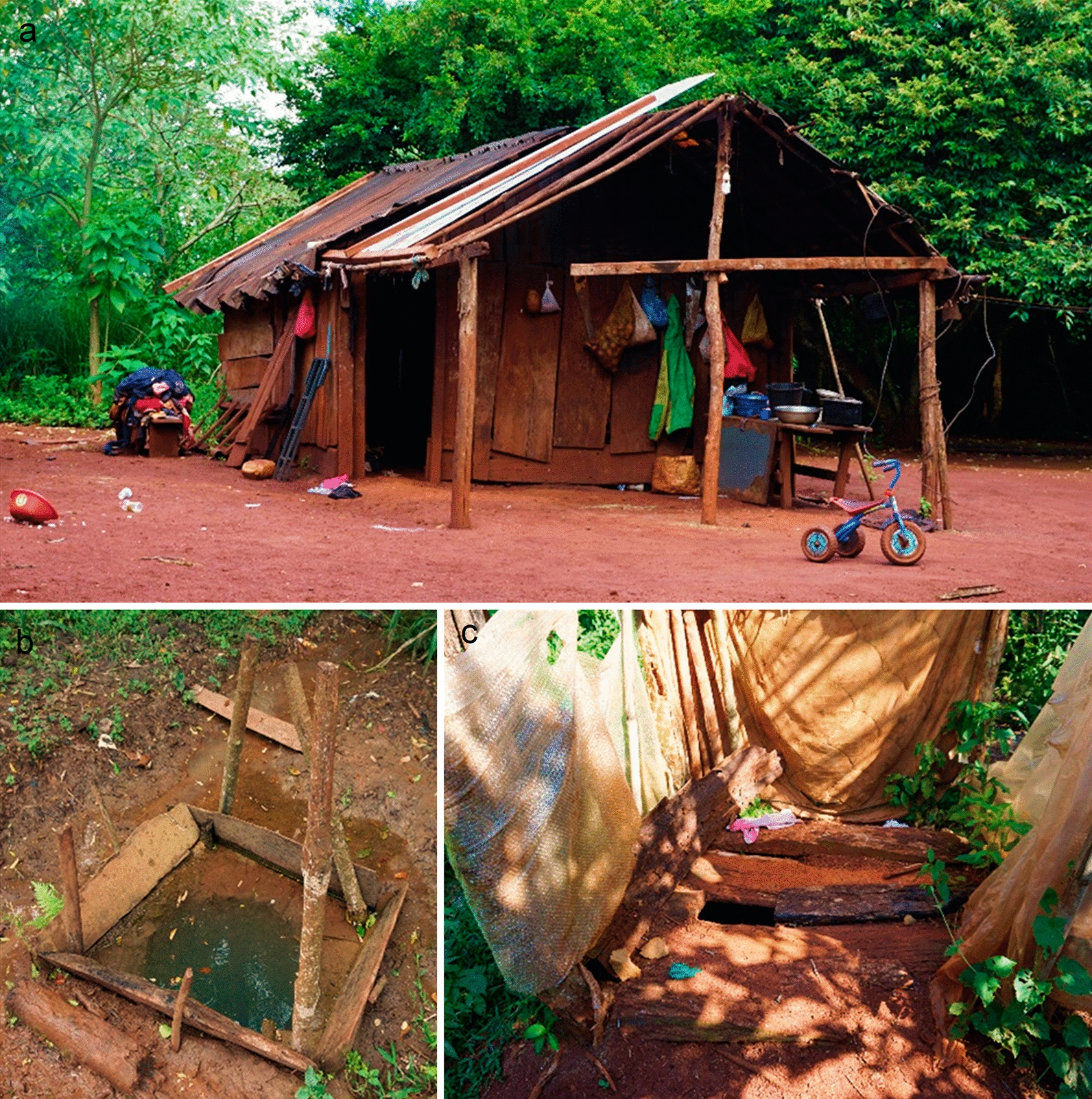
An example of a household (**a**), a water well (**b**) and a latrine (**c**) in the Fortin Mbororé Village, Puerto Iguazú, Misiones (Argentina)

Around the city’s periphery, Mbyá Guaraní aboriginal communities have settled into villages, including the village of Fortín Mbororé, which is composed of around 200 families. The primary source of income is from guided tours organized to visit the village, handcrafts and social plans. These communities also all share similar water and sanitation conditions and are homogeneous in their economic status, with low monthly incomes [16]. The living conditions in Fortín Mbororé are characterized by a lack of water and sanitation [16] and houses made of adobe bricks with unimproved roofs and dirt floors, and practically the entire population, both children and adults, walk barefoot (Fig. 2). Most houses have a single room for sleeping, and therefore overcrowding is common.

### Study design

A cross-sectional study was conducted in Fortín Mbororé between January and March 2018, as a community-wide intervention. The study included individuals older than 1 year. A total of 61 households were visited, georeferenced and characterized using a house-by-house questionnaire, collecting data on all members of the household, including socioeconomic variables; these are detailed in the results (Table 4).

Oral bilingual (Spanish/Mbyá-Guaraní) explanations on sample collection were provided along with sample collection containers without any fixative [one per person], and were retrieved on the following day. The fresh samples were transported without fixative in a refrigerated icebox and kept at 4 °C in the lab until analysis within 24 h of collection.

Together with stool samples, blood samples were drawn though venipuncture into H2PP tubes (VITIS^®^, Prunus SLR, Argentina) containing EDTA-K3 anticoagulant and transported to a private clinical laboratory (Clínica SAM) located in Puerto Iguazú for hematological analysis. A complete hemogram was performed, including hemoglobin values (Hgb), white blood cell counts, eosinophil relative count and hematocrit. Subjects were classified as anemic or not anemic using the thresholds to define anemia according to sex and age as defined by WHO [28]. All participants were offered anthelminthic treatment according to the WHO preventive chemotherapy for human helminthiasis [29] and national guidelines [30], with the inclusion of ivermectin (200 µg/kg), together with WASH [water, sanitation, and hygiene] education workshops to improve hygiene habits.

### Stool examination

To determine the presence of intestinal parasites, samples were processed with four different diagnostic techniques [31–33] to optimize the detection of a diverse parasite spectrum. The techniques used included the Ritchie concentration technique for detection of both protozoan and helminth parasites, Baermann concentration for the detection of larvae, Kato–Katz to measure infection intensity of helminth parasites, and modified Ziehl–Neelsen stain for detection of coccidia. The Kato–Katz technique was used only if eggs or larval stages were previously detected by the Ritchie or Baermann method; results were recorded as eggs per gram (EPG) and classified as light, moderate or heavy infection following WHO guidelines [34]. Aliquots of the fresh samples were stored either in 10% formalin for confirmatory techniques or at −20 °C with no fixative for molecular biology techniques. If the sample volume was insufficient to perform all diagnostic methods, the sedimentation technique was prioritized due to its overall higher sensitivity [35]. The findings from each of the different methods were recorded in a database.

### DNA extraction

Genomic DNA was extracted from 200 mg of concentrated fecal material using the QIAamp DNA Stool Mini Kit (QIAGEN, Hilden, Germany) according to the manufacturer’s instructions, with slight modification; fecal samples were mixed with stool lysis buffer and incubated for 10 min at 95 °C. The DNA was eluted in 100 μl of elution buffer and stored at −20 °C.

### Molecular identification of *Giardia* spp., *Blastocystis* spp. and *Entamoeba* spp.

PCR reactions were performed using an MJ Mini Thermal Cycler PTC-1148 (Bio-Rad Laboratories, Inc.). Sterile water was used as a negative PCR control, and previously tested fecal samples containing *G. intestinalis*, *Blastocystis* spp., *E. histolytica* and *E. dispar* were used as positive controls. The oligonucleotides used for molecular identification and characterization of *G. intestinalis*, *Blastocystis* spp. and *Entamoeba histolytica/dispar* appear in Additional file 1: Table S1. PCR products from all of the reactions were run on a 1% agarose gel, except for the PCR products for *Blastocystis* spp., which were run on a 2% agarose gel.

### Molecular detection of *Giardia intestinalis*

Samples positive for *Giardia* spp. through microscopy were screened by a quantitative PCR (qPCR) method targeting a specific 62-bp region of the small subunit rRNA (SSU rRNA) gene of the parasite [36]. Amplification reactions were conducted in total volumes of 25 µl with 3 µl template DNA, 12.5 pmol of primers and 1× TaqMan Gene Expression Master Mix (Applied Biosystems, CA, USA). Reactions were run using the following protocol: an initial hold step of 2 min at 60 °C, 10 min at 95 °C and 45 cycles of 15 s at 95 °C and 1 min at 60 °C.

### Molecular typing of *G. intestinalis*

*Giardia intestinalis* isolates that tested positive by qPCR with cycle threshold values less than 37 (Ct < 37) were genotyped to assemblage level using a nested PCR encoding a 753-bp fragment of the β-giardin (*bg*) gene of the parasite [37, 38]. In general, PCR mixtures (25 µl final volume) consisted of 8.5 µl of MyTaq Reaction Buffer, containing 5 mM dNTPs and 15 mM MgCl_2_, 2.5 units (U) of MyTaq DNA polymerase (Bioline GmbH, Luckenwalde, Germany), 1 µl of each 10 µM primer pair and 5 µl of extracted DNA for the first PCR reaction. The amplification condition for the first PCR reaction was as follows: initial denaturation at 95 °C for 7 min, followed by 35 cycles (95 °C for 30 s, 65 °C for 30 s and 72 °C for 60 s), and the final extension was at 72 °C for 7 min. For the second PCR reaction, 3 µl of the product from the first PCR reaction was added, and the reaction was performed under the same conditions as above except for the cycling time, which was instead 95 °C for 30 s, 55 °C for 30 s and 72 °C for 60 s.

### Molecular typing of *Blastocystis* spp.

Characterization of the *Blastocystis* subtypes from the microscopic-positive samples was achieved by PCR, targeting the SSU rRNA gene of the parasite, amplifying a PCR product of ~ 600 bp [39]. The reaction mixture (25 µl) contained 2.5 U of MyTaq DNA polymerase (Bioline GmbH, Luckenwalde, Germany), 5× MyTaq Reaction Buffer, 5 μl of template DNA and 0.5 μM of each primer. Amplification conditions consisted of one step of 95 °C for 3 min, followed by 30 cycles of 1 min each at 94 °C, 59 °C and 72 °C, with an additional 2 min final extension at 72 °C.

### Molecular detection of *Entamoeba histolytica*/*dispar*

*Entamoeba histolytica/dispar* (*Entamoeba* complex) are morphologically identical species. In this study, specific primers were used to identify either *E. histolytica* or *E. dispar*. To identify a 166-bp product for *E. histolytica* and a 752-bp product *E. dispar* DNA, samples were screened using a specific PCR based on SSU rRNA [40]. The reaction mixture contained 5 µl of DNA, 1.25 µl of each primer, 2.5 U of Taq polymerase (MyTaq DNA polymerase, Bioline GmbH) and 5× MyTaq Reaction Buffer containing 5 mM dNTPs. PCR amplification started with an initial denaturation at 94 °C for 3 min, followed by 30 cycles of 94 °C 1 min, 58 °C for 1 min and 72 °C for 1 min, with a final extension at 72 °C for 7 min.

### Sequencing and phylogenetical analysis

All PCR amplicons obtained from *G. intestinalis*-positive samples were purified using an mi-PCR Purification Kit (Metabion International AG, Martinsried, Germany) and were sent for sequencing in both direction using the corresponding internal primers to the Central Service for sequencing for Experimental Research (SCSIE, University of Valencia, Spain). β-giardin DNA sequences, both forward and reverse directions, obtained from *G. intestinalis*-positive samples, were viewed using the Chromas 2.6.6v (Technelysium Pty Ltd.) sequence analysis program. The BLASTn tool (http://blast.ncbi.nlm.nih.gov/Blast.cgi) was used to compare β-giardin nucleotide sequences with sequences retrieved from the NCBI GenBank database. Generated DNA consensus sequences from the β-giardin fragment were aligned using the ClustalW algorithm, and a phylogenetical tree was constructed using the neighbor-joining method in MEGA X version 10.1.7 (Molecular Evolutionary Genetics Analysis) software. The reliability of the phylogenetic tree at each branch node was estimated by the bootstrap method using 500 replications. Reference β-giardin sequences chosen for comparison were from isolates of human and animal origin collected throughout the world and from Italian clinical samples, which are described in Additional file 2: Table S2. *Giardia muris* was used as the outgroup.

*Blastocystis* sequences were submitted to the *Blastocystis* 18S database (http://pubmlst.org/blastocystis/) for subtype confirmation and allele identification.

### Statistical analysis

Data were analyzed using Stata 14.2 software (STATA Corp., TX, USA). Measures were evaluated using proportions with 95% confidence intervals (95% CI) and means with standard deviations (SD). The Chi-square test was used to compare significant associations between different variables. Associations were obtained comparing presence or absence of protozoa and helminth (by age group and by parasitic species). A probability (*P*) value < 0.05 was considered as evidence of statistical significance. Odds ratios (OR) with 95% confidence intervals (CI) were used to measure the strength between dependent and independent variables.

## Results

### Study population

In total, 218 individuals from 61 households provided stool samples. Population distribution was 47.7% male (*n* = 104) and 52.3% female (*n* = 114), with a mean age of 19.9 years and a range from 1 to 87 years. Individuals younger than 15 years represented half of the participants (50.9%). Over half of the underage population were in school (57%), and 76% of adults had gone to school, although not all of them had completed their education; 72.6% had attended primary school and only 24.7% high school. Nonetheless, more than half of the population (64.0%) knew how to read and write.

### Prevalence of intestinal parasites

The stool samples from all 218 individuals were analyzed through the Ritchie sedimentation technique. Two hundred and three samples had enough material for the Baermann concentration technique, and only those positive for either one of the previous techniques were processed by the Kato–Katz technique (*n* = 134) in order to quantify helminth eggs. Additionally, 209 of the samples were processed using the modified Ziehl–Neelsen stain. Due to the small amount of fecal material received from nine of the 218 samples, only the Ritchie technique was performed.

The overall prevalence of intestinal parasites was 92.7% (202/218), including 82.6% protozoans and 78.4% helminths (Table 1). The most prevalent protozoans were *Blastocystis* spp. (57.3%), followed by *Entamoeba coli* (41.7%) and *G. intestinalis* (24.8%), while the most prevalent helminth was hookworm (72%). There was also an 11.5% prevalence of *S. stercoralis*. *Cryptosporidium* spp. infection was not detected in any of the analyzed samples, and *Enterobius vermicularis* was practically undetected. Polyparasitism was frequent (88.1%), mainly with two or three parasite species (29.8%), with one sample harboring up to eight parasites (Table 1). The most common co-infections found were *E. coli* + *Blastocystis* + hookworm (*n* = 47) and *Blastocystis* + *G. intestinalis* + hookworm (*n* = 24) (Table 1).

**Table 1:** Prevalence of intestinal parasites and polyparasitism in individuals (*n* = 218) from Fortín Mbororé Village (Puerto Iguazú, Misiones, Argentina)

Parasite	*N*	% Prevalence [95% CI]
Protozoa	180	82.6 [76.9–87.1]
* Entamoeba coli*	91	41.7 [35.1–48.3]
* Entamoeba complex*	22	10.1 [6.1–14.1]
* Entamoeba hartmanni*	31	14.2 [9.5–18.9]
* Endolimax nana*	45	20.6 [15.2–26.1]
* Iodamoeba bütschlii*	11	5.0 [2.1–8.0]
* Chilomastix mesnili*	12	5.5 [2.3–8.6]
* Giardia intestinalis*	54	24.7 [19–30.5]
Protist	125	57.3 [50.7–64.0]
* Blastocystis* spp.	125	57.3 [50.7–64.0]
Helminths	171	78.4 [72.9–83.9]
* Enterobius vermicularis*	3	1.4 [0.2–2.9]
* Hymenolepis nana*	34	15.6 [10.7–20.5]
* Trichuris trichiura*	1	0.5 [0.4–1.4]
* Ascaris lumbricoides*	3	1.4 [0.2–2.9]
Hookworms	157	72.0 [66.0–78.0]
* Strongyloides stercoralis*	25	11.5 [7.2–15.7]
Total positives	202	92.7 [88.3–95.5]
Polyparasitism	178	88.1 [82.8–91.9]
Double	53	29.8 [23.5–37.0]
Triple	53	29.8 [23.5–37.0]
Quadruple	45	25.3 [19.4–32.2]
Quintuple	19	10.7 [6.9–16.2]
Sextuple	7	3.9 [1.9–8.1]
Octuple	1	0.5 [0.1–3.9]

*CI* confidence interval

*Giardia intestinalis* and *C. mesnili* were the most frequent IPIs among preschool-age children (*χ*^2^ = 18.83, *df* = 1, *P* = 0.001), while *Hymenolepis nana* and *Blastocystis* spp. were the most frequent among school-age children (*χ*^2^ = 20.92, *df* = 1, *P* = 0.007). Hookworm was the most frequent IP found in adults (*χ*^2^ = 9.29, *df* = 1, *P* = 0.007), while *S. stercoralis* was the most frequent among the female gender (*χ*^2^ = 4.39, *df* = 1, *P* = 0.003). There was an association (*χ*^2^ = 4.69, *df* = 1, *P* = 0.035) between no formal education and the presence of intestinal parasites (Table 2).

**Table 2:** Descriptive characteristics of participants from Fortín Mbororé, Puerto Iguazú (Misiones, Argentina) and prevalence of intestinal parasites (IPs)

Characteristics	Total participants (*n* = 218)	Presence of IP (*n* = 202)	Absence of IP (*n* = 16)	Percentage of infected per characteristic [95% CI]	*P*-value
Age group (years)					
1–5	44	36	8	81.8 [66.9–90.9]	*0.002*^a^
6–12	54	53	1	98.1 [87.3–99.8]	0.075^b^
13–19	26	25	1	96.2 [74.6–99.5]	0.467
19–27	33	30	3	90.0 [74.1–97.2]	0.675^c^
> 27	61	58	3	95.1 [85.4–98.5]	0.393^c^
Gender					
Female	114	107	7	93.6 [87.6–97.1]	0.477^d^
Male	104	95	9	91.3 [84.0–95.5]	0.477
Education					
In preschool	3	3	0	100	0.718
In elementary school	82	78	4	95.1 [87.5–98.2]	0.280
In high school	19	17	2	89.5 [62.9–97.7]	0.577
Finished elementary school	27	26	1	96.3 [75.5–99.5]	0.439
Incomplete elementary school	3	3	0	100	0.718
Finished high school	9	9	0	100	0.724
No formal education	69	60	9	86.9 [76.5–93.2]	*0.035*

Values in italic indicate the relationship was statistically significant *P* < 0.05

*CI* confidence interval

^a^*G. intestinalis* and *C. mesnili* *P* = 0.001 at this age group

^b^*H. nana* and *Blastocystis* spp. *P* = 0.007 at this age group

^c^Hookworm *P* = 0.007 at this group

^d^*S. stercoralisP* = 0.03 at female group

**Fig. 3:**
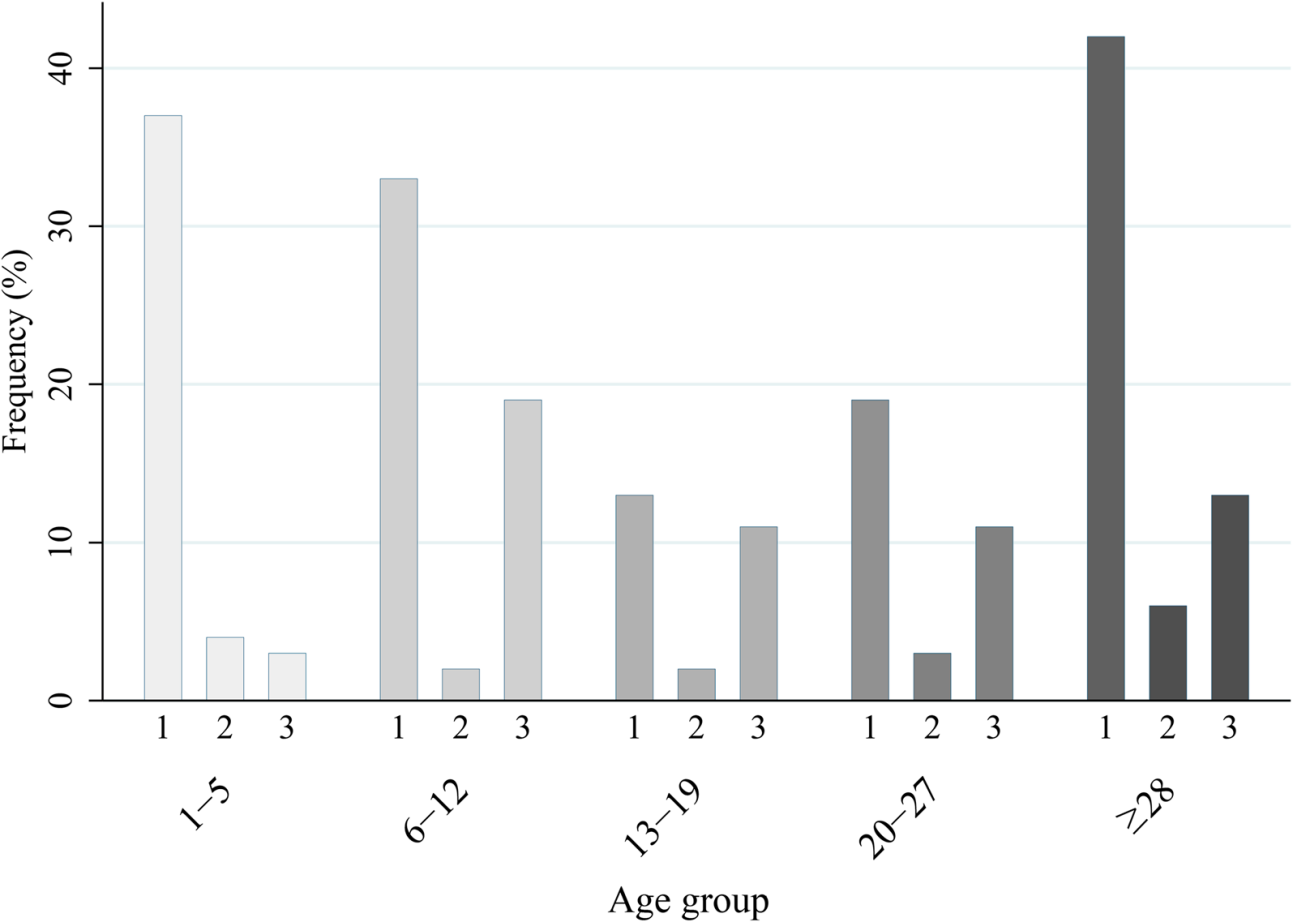
Intensity of hookworm infection by age group. Number of participants from Fortin Mbororé, Puerto Iguazú (Misiones, Argentina) with light (1), moderate (2) or heavy (3) hookworm infection, by age group (1–5, 6–12, 13–19, 20–27, and 28 years of age or older)

The intensity of hookworm, measured as EPG of feces using the Kato–Katz technique, was mostly of light (66.1%) and heavy (26.2%) infection, and no statistically significant differences were found either between age groups or by sex. The distribution of hookworm infection intensity by age group is reflected in Fig. 3, which shows the frequency of light, medium or heavy infection within each age group. The intensity of the only *T. trichiura* infection found was light (48 EPG), while the *A. lumbricoides* infections detected were of light and heavy intensity (168, 408 and 14,352 EPG, respectively).

### Intestinal parasites and anemia

Approximately 72.4% of the population presented with anemia, although females were significantly more affected (*χ*^2^ = 6.57, *df* = 1, *P* = 0.010). Only hookworm infections were significantly associated with anemia (OR = 1.97; 95% CI 0.9–4.3), with statistical association for male sex (*P* = 0.02; OR = 3.80; 95% CI 1.20–12.04). No association between the presence of anemia and education level was found (Table 3).

**Table 3:** Association between sex, age and soil-transmitted helminths (STH) and presence of anemia in participants from Fortín Mbororé Village (Puerto Iguazú, Misiones, Argentina)

	Presence of anemia (*N*)		Percentage (%) [95% CI]	*P*-value
	Yes	No		
Population	118	45	72.4 [64.9–78.8]	
Sex				
Male	47	28	62.7 [51.0–73.0]	
Female	71	17	80.7 [70.9–87.8]	*0.010*
Age group				
1–5	11	10	52.4 [30.2–73.7]	
6–12	30	9	76.9 [60.5–87.9]	
13–19	17	5	77.3 [53.5–90.9]	0.116
19–27	26	5	83.9 [65.4–93.5]	
> 27	34	16	68 [53.5–79.7]	
Hookworm				
Male				
Yes	41	18	69.5 [56.3–80.1]	*0.019*
No	6	10		
Female				
Yes	55	13	80.9 [69.5–88.7]	0.930
No	16	4		
*S. stercoralis*				
Male				
Yes	2	2	50 [24.7–97.5]	0.590
No	45	26		
Female				
Yes	11	3	78.6 [46–94]	0.830
No	60	14		

Values in italic indicate the relationship was statistically significant *P* < 0.05

*CI* confidence interval

### Household characteristics

The main characteristics from the houses and their relationship with the presence of intestinal parasites are detailed in Table 4. Most of the inhabitants live in overcrowded houses (68.4%), generally with only one room for the whole family. An association between the presence of intestinal parasites and overcrowding (*χ*^2^ = 7.62, *df* = 1, *P* = 0.005) was found, with the odds of having intestinal parasites increased up to four times. The employment situation of the families was precarious, and the main livelihoods were animal farming (53.9%) and crafts (71.6%), while some families benefited from social plans (27.4%). Almost all the families (90.4%) had at least one dog, so practically the entire population lived with animals in their environment (97.2%).

**Table 4:** Household characteristics and their association with the presence or absence of intestinal parasites in Fortín Mbororé, Puerto Iguazú, Misiones, Argentina

Characteristics	Presence of IP (*n* = 199)	Absence of IP (*n* = 16)	OR (95% CI)	*P*-value
Animal farming				
Yes	175	12	2.43 (0.7–8.1)	0.139
No	24	4	0.41 (0.1–1.4)	
Water source				
Borehole	125	12	0.56 (0.2–1.8)	0.329^a^
Tap water	74	4	1.776 (0.6–5.7)	
Overcrowding				
Yes	141	6	4.05 (1.4–11.6)	*0.005*
No	58	10	0.25 (0.1–0.7)	
Wall				
Improved (wood)	165	14	0.7 (0.2–3.3)	0.479
Unimproved	34	2	1.4 (0.3–6.5)	

	Presence of STH (*n* = 137)	Absence of STH (*n* = 45)		
Floor				
Cement	33	24	0.28 (0.1–0.6)	*0.001*
Wood	24	8	0.9 (0.4–2.4)	0.982
Dirt floor	80	13	3.5 (1.7–7.2)	*0.001*
Latrine				
Yes	110	40	0.5 (0.2–1.4)	0.189
No	27	5	1.96 (0.7–5.4)	
Barefoot				
Yes	119	40	1.16 (0.5–2.9)	0.762
No	18	7	0.9 (0.3–2.2)	

Values in italic indicate the relationship was statistically significant *P* < 0.05

^a^*E. histolytica/dispar* associated with tap water source (*P* = 0.020)

Most of the houses were made of wooden walls (83.3%) or thin wooden panels (55.8%) and dirt floors (50.7%). This type of floor was associated with increased transmission of STH (*χ*^2^ = 15.02, *df* = 1, *P* = 0.001), and decreased by 72.2% (0.278, 95% CI 0.1–0.6, *P* = 0.001) for those living in houses with cement floors. Additionally, 87.6% of the participants walked barefoot and 16.2% practiced open defecation, although most had a latrine with simple ground excavation (79.1%). Nonetheless, no significant associations were observed between these factors and infection with IPs.

The most prevalent protozoan parasites observed were those transmitted by the fecal–oral route and through water. Most of the community (63.7%) obtained water from boreholes, and the rest of the families used tap water. None of the families stated that they treated the water for drinking or cooking by either boiling or use of disinfectants (i.e. bleach). There was no association between infection with protozoans as a group and water source, but families drinking from tap water had a higher prevalence of waterborne parasites. When analyzed by protozoan species, infection with *E. histolytica/dispar* was significantly associated with the use of tap water (*χ*^2^ = 4.39, *df* = 1, *P* = 0.020).

### Molecular characterization of *G. intestinalis* isolates

Through the standard coprological techniques used, 54 samples were found to be positive for *Giardia* spp. Of these, only those with a high number of cysts/slide were selected for molecular analysis (*n* = 32), and 29 of the 32 DNA isolates tested positive for *G. intestinalis* by qPCR. Generated Ct values ranged from 28.3 to 39.1 (median: 32.2; 25th centile: 30.5; 75th centile: 34.0). Only DNA isolates with Ct values ≤ 37 (*n* = 28) were used for genotyping.

**Fig. 4:**
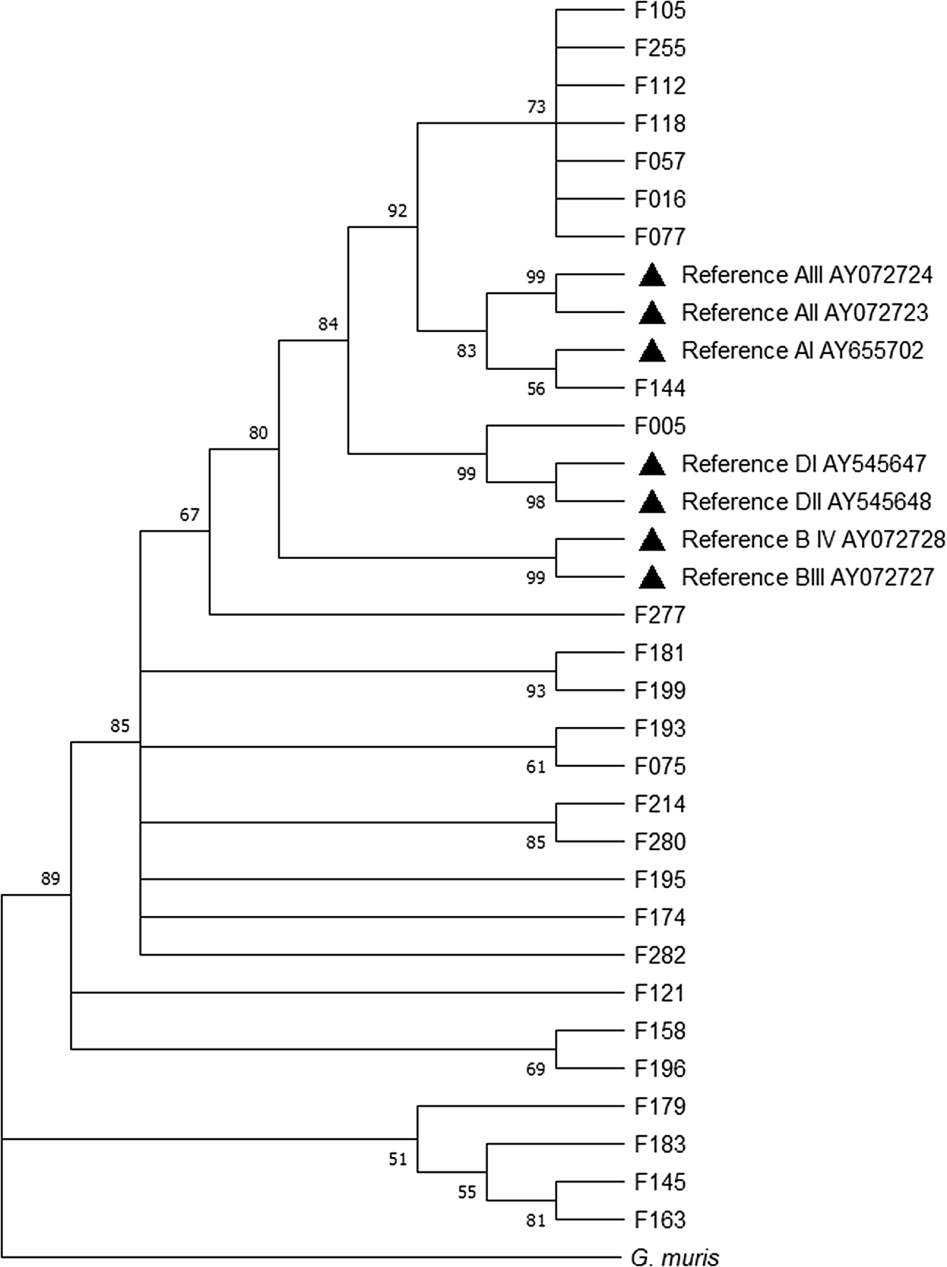
Phylogenetic relationships of *Giardia intestinalis* inferred by neighbor-joining analysis of the β-giardin nucleotide sequences. Filled triangles represent reference sequences obtained from GenBank, described in Additional file 2: Table S2. A sequence from *Giardia muris* was used as the outgroup. Bootstrap values are based on 500 replicates, and only bootstraps > 50% are indicated

Of the 28 DNA isolates selected, 92.9% (26/28) were successfully amplified at the β-giardin locus and are described in Additional file 3: Table S3. Sequence analyses revealed the presence of assemblages A (30.8%; 8/26) and B (65.4%; 17/26); one canine assemblage (D) was also detected. Type A assemblage sequences were assigned to either sub-assemblage AII (12.5%; 1/8) or AIII (87.5%; 7/8), while all type B sequences were assigned to the BIV sub-assemblage. The phylogenetic analysis performed with the sequences obtained revealed that the sample sequences found herein clustered with the corresponding assemblage and sub-assemblage A–D sequences used as references, although some samples tended to group into independent subgroups, reflecting noticeable changes at the nucleotide level (Fig. 4).

### Molecular characterization of *Blastocystis* isolates

**Fig. 5:**
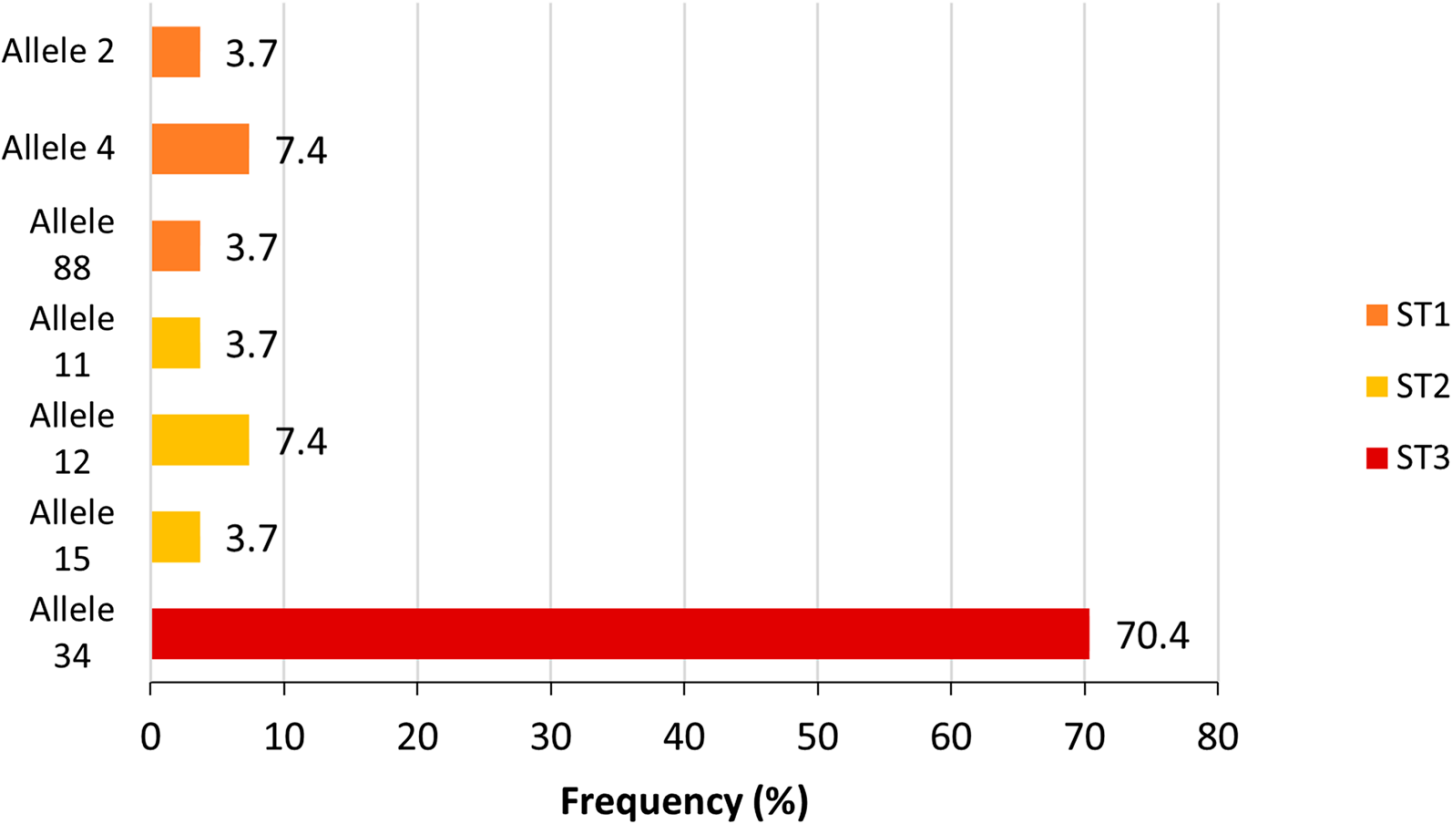
Diversity and frequency of *Blastocystis* spp. subtypes and 18S alleles identified from positive samples, identified through microscopy, of participants from Fortín Mbororé (Puerto Iguazú, Misiones, Argentina)

Through the standard coprological techniques used, 125 samples were found to be positive for *Blastocystis* spp.; only those with high numbers of cyst/slide were selected for molecular analysis (*n* = 66). After rejecting unreadable or poor-quality sequences typically associated with faint bands on agarose gels, 27 isolates were successfully subtyped (40.9%) and are detailed in Fig. 5. Sequence analysis at the SSU rDNA (barcode region) gene of the parasite revealed the presence of three subtypes (ST): ST1 (14.8%; 4/27), ST2 (14.8%, 4/27) and ST3 (70.4%; 19/27). Three different alleles were observed for ST1 (2, 4 and 88), three for ST2 (11, 12, 15), and a single allele (34) for ST3.

### Molecular characterization of *E. histolytica/dispar*

Through the standard coprological techniques used, 22 samples were found to be positive for the “*Entamoeba* complex” and processed by PCR. Molecular characterization of the isolated samples showed that 45.5% of them were positive for *E. histolytica*, and *E. dispar* was not identified in any of the samples.

## Discussion

The overall prevalence of IPs found in individuals from Fortín Mbororé Village, located in Puerto Iguazú, Misiones, Argentina was 92.7%, with a high prevalence of both protozoa and helminths. The most prevalent pathogenic protozoans were *G. intestinalis* (24.7%) and *Blastocystis* spp. (57.3%), whose pathogenic capacity is still a debatable issue. The most prevalent STH was hookworm (72%), followed by *S. stercoralis* with a prevalence of 11.5%. The percentage of individuals with polyparasitism was higher than 88%, evidencing the need for an urgent improvement of the health and living conditions of the population. These results are similar to previous studies carried out in other areas from northern Argentina, in populations who presented higher socio-environmental vulnerability [5, 15].

In the multivariate analysis of the data, a higher presence of IPs was observed in preschool- and school-age children. Also, a significant relation between no formal education and a higher presence of IPs was observed, as previously reported [41]. In contrast, parasitism was not associated with gender. The high prevalence of parasites transmitted by the oral–fecal route suggests that water quality is not adequate. Furthermore, the population that obtained water from the public water system was positively associated with infection by *E. histolytica/dispar* and had a higher probability of harboring a parasitic infection (OR = 1.77). Some of the household characteristics of the study area, such as dirt floor or overcrowding, were associated with a higher prevalence of STH or IPs, as observed in other studies [7, 42]. Unimproved sanitation, walking barefoot or practicing open defecation are risk factors that may contribute to an increase in skin-penetrating parasite infections like hookworm. In the present study, households with cement floors showed lower incidence of STH, so an initial improvement of the material conditions of the houses might have an impact on the prevalence of this type of parasite, which would theoretically decrease even more with the use of footwear.

The high prevalence of hookworm and *S. stercoralis* detected in Iguazú coincides with rates reported previously [4, 15], although a large difference in prevalence was observed for *S. stercoralis* (41.9% vs. 11.5%). These differences may be due to the variability in parasite expulsion in the stool and the low sensitivity and detection limitations of the nonmolecular techniques used [19, 43]. There were practically no cases of *A. lumbricoides* or *T. trichiura*; these parasites are more influenced by fecal–oral hygiene habits, and tap water and hand washing could contribute to a lower prevalence detected in our study [6]. Although studies investigating risk factors for *S. stercoralis* infection have mostly reported a higher risk among men, generally attributed to men’s extensive exposure to soil during farming activities [44], in our study this parasite was associated more with females. We can only hypothesize on the reason for this, although it is important to note that there was no bias in sampling, since the participation between males and females was not statistically different. One plausible explanation is that men in this area do not perform farming activities; they usually travel outside the village to tourist areas to sell their crafts, while women mostly stay at home to care for the children.

Additionally, 72.4% of the population studied was anemic. The relationship between STH infection, malnutrition and anemia has been extensively shown [15]. In this study, a significant association was observed between the presence of hookworm and anemia in men. Furthermore, the intensity of hookworm infection is related to anemia and morbidity and is a key indicator for measuring the success of large-scale deworming programs [45, 46]. Also, deleterious nutritional conditions and protein malabsorption have been associated with elevated parasite loads [15]. However, no association was found between heavy intensity (26.2%) and a greater presence of anemia. This is probably because most of the population studied had light-intensity hookworm infections (66.1%). The low nutritional status of the population previously reported [15] could be influenced, among other factors, by parasite incidence, since, as the study points out, these two factors are associated. Guidelines for anemia and STH recommend implementing deworming programs to improve the public health situation of communities with high prevalence of anemia [10, 47].

Infection with *E. histolytica* can result in invasion of the colon wall and damage to other host tissues (amebiasis), and remains a cause of morbidity and mortality in developing countries [48]. The clinical diagnosis of amebiasis is usually confirmed by visualization of the parasite by light microscopy, but this has the limitation of being unable to distinguish *E. histolytica* from *E. dispar* and *E. moshkovskii* cysts. Furthermore, the presence of other *Entamoeba *spp.,* Iodamoeba* spp. or *Endolimax* spp. can make the diagnosis even more difficult [40]. With the aid of molecular techniques, it was possible to identify 10 *E. histolytica*-infected individuals in 22 microscopy-positive samples. The remaining 12 negative samples by PCR suggest they are cysts that may belong to another *Entamoeba* spp. The low prevalence of this protozoan in the current study agrees with previous studies from northern Argentina [49, 50].

On the other hand, *Blastocystis* spp. was the most prevalent species detected in this study, which is a zoonotic parasite that colonizes humans and multiple domestic and wild animals. The high prevalence contrasts greatly with data obtained in another study on the same population (57.3% vs. 5.9%) [15], although another study conducted in the Mbyá-Guaraní communities (Misiones, Argentina) has reported a higher prevalence [4]. The genetic diversity of *Blastocystis* spp. can be classified based on their polymorphic regions of its small subunit of the ribosomal RNA gene [51]. Some studies have reported that the most frequent subtypes in humans in Latin America are ST1 and ST3 [22, 25]. Although its immunopathogenesis is a matter of study and controversy, the ST3 subtype has been associated with a higher frequency in symptomatic patients [52]. This first molecular approximation of *Blastocystis* spp. in Iguazú indicates that the majority of the population was infected with the ST3 (87%) subtype, although gastrointestinal symptoms were not the subject of an associative analysis in the current study. Considering the zoonotic nature of the parasite, found in almost 60% of the population, and the fact that practically all families live with domestic or farm animals, these may represent a significant zoonotic source of this parasite for the community of Fortín Mbororé.

With respect to *G. intestinalis*, assemblage B showed a higher prevalence (65.4%) compared to assemblage A (30.8%); sub-assemblages AIII and BIV were the most frequent. These results coincide with the prevalence reported in other studies [24, 53], and it is commonly believed that humans are infected only with assemblages A and B. Surprisingly, a case of infection by assemblage D (identified from dogs) was found in this study, coinciding with the recent identification of unusual *G. intestinalis* genotypes, such as assemblages C, D, E and F in humans [54–56]. These results indicate the need to carry out greater genotyping of *G. intestinalis* infections in order to increase our knowledge on the transmission routes of this parasite.

## Conclusion

This study represents the first molecular approach in Puerto Iguazú, describing the genotype of *G. intestinalis, Blastocystis* spp. and molecular detection of *E. histolytica*. There was a significant inverse association between age and parasitism, and a higher prevalence of IPs was associated with no formal education and with poor household characteristics. Domestic animals were found to be implicated in the zoonotic transmission of *Giardia* spp. and *Blastocystis* spp. The protozoans detected in the population are transmitted through water contaminated with fecal matter, evidencing the need to improve the quality of water and to improve access to appropriate sanitation. A hyperendemic area for STH was found, with hookworm infections associated with anemia. Mass deworming programs, together with WASH and health education, need to be implemented in this area to control and decrease the prevalence of IPs in general and STH in particular.

## Supplementary Information

**Additional file 1: Table S1.** Oligonucleotides used for the molecular identification and characterization of *Giardia intestinalis*, *Blastocystis* spp. and *Entamoeba histolytica/dispar* in this study.

**Additional file 2: Table S2.** Reference sequences of *Giardia intestinalis* used in the comparative analysis to build the phylogenetic tree.

**Additional file 3: Table S3.** List of *Giardia intestinalis*-positive samples from participants of Fortín Mbororé, Puerto Iguazú, Misiones (Argentina) identified by microscopy and qPCR that were also identified at the assemblage level using the β-giardin gene.

## Data Availability

Data supporting the conclusions of this article are included within the article. The datasets used and/or analyzed during the current study are available from the corresponding author on reasonable request.

## Abbreviations

*bg*: β-Giardin
CI: Confidence interval
Ct: Cycle threshold
EPG: Eggs per gram
Hgb: Hemoglobin
IDA: Iron-deficiency anemia
IP: Intestinal parasites
IPI: Intestinal parasite infection
NCBI: National Center for Biotechnology Information
OR: Odds ratio
qPCR: Quantitative real-time polymerase chain reaction
SD: Standard deviation
SSU rRNA: Small subunit ribosomal RNA
ST: Subtypes
STH: Soil-transmitted helminths
WASH: Water, sanitation and hygiene
WHA: World Health Assembly
WHO: World Health Organization
