# Supplementary material for: Prevalence of intestinal parasites and molecular characterization of Giardia intestinalis, Blastocystis spp. and Entamoeba histolytica in the village of Fortín Mbororé (Puerto Iguazú, Misiones, Argentina)
Source: Parasit Vectors. 2021 Oct 1;14:510. doi: 10.1186/s13071-021-04968-z (PMC8485468; doi:10.1186/s13071-021-04968-z)
Supplement: Supplementary file 1 — Additional file 1: Table S1. Oligonucleotides used for the molecular identification and characterization of Giardia intestinalis, Blastocystis spp. and Entamoeba histolytica/dispar in this study. [file 13071_2021_4968_MOESM1_ESM.docx]

**Additional file 1: Table S1.** Oligonucleotides used for the molecular identification and characterization of *Giardia intestinalis*, *Blastocystis* spp. and *Entamoeba histolytica/dispar* from human fecal samples collected during the study conducted in Fortín Mbororé Village (Puerto Iguazú, Misiones, Argentina).

| Target organism | Gene fragment | Oligonucleotide | Sequence (5´–3´) | Reference |
| --- | --- | --- | --- | --- |
| *Giardia intestinalis* | SSU rRNA | Primers | CCCGCGGCGGTCCCTGCTAG | [36] |
|  |  | Gd-80F | GACGGCTCAGGACAACGGTT | [36] |
|  |  | Gd-127R | TTGCCAGCGGTGTCCG | [36] |
|  | β-giardin | G7-F | AAGCCCGACGACCTCACCCGCAGTGC | [37] |
|  | β-giardin | G759-R | GAGGCCGCCCTGGATCTTCGAGACGAC | [37] |
|  | β-giardin | G99-F | GAACGAACGAGATCGAGGTCCG | [38] |
|  | β-giardin | G609-R | CTCGACGAGCTTCGTGTT | [38] |
| *Blastocystis* spp. | SSU rRNA | RD5-F | ATCTGGTTGATCCTGCCAGT | [39] |
|  |  | BhRD-R | GAGCTTTTTAACTGCAACAACG | [39] |
| *Entamoeba histolytica/dispar* | SSU rRNA | Enta-F | ATGCACGAGAGCGAAAGCAT | [40] |
|  |  | Eh-R | GATCTAGAAACAATGCTTCTCT | [40] |
|  |  | Ed-R | CACCACTTACTATCCCTACC | [40] |
