# Supplementary material for: Prevalence of intestinal parasites and molecular characterization of Giardia intestinalis, Blastocystis spp. and Entamoeba histolytica in the village of Fortín Mbororé (Puerto Iguazú, Misiones, Argentina)
Source: Parasit Vectors. 2021 Oct 1;14:510. doi: 10.1186/s13071-021-04968-z (PMC8485468; doi:10.1186/s13071-021-04968-z)
Supplement: Supplementary file 2 — Additional file 2: Table S2. Reference sequences of Giardia intestinalis used in the comparative analysis to build the phylogenetic tree. [file 13071_2021_4968_MOESM2_ESM.docx]

**Additional file 2: Table S2.** Reference sequences of *Giardia intestinalis* used in this study to compare with the sequences obtained from the participants from Fortín Mbororé Village, Puerto Iguazú (Misiones, Argentina) in order to build the phylogenetic tree and determine the assemblages and sub-assemblages circulating in the community.

| Species | GeneBank accession number | Assemblage | Sub- assemblage | Host | Country | Reference |
| --- | --- | --- | --- | --- | --- | --- |
| *Giardia intestinalis* | AY655702 | A | A I | *Bos Taurus* | USA | [41] |
|  | AY072723 | A | A II | Human | Italy | [37] |
|  | AY072724 | A | A III | Human | Italy | [37] |
|  | AY072727 | B | B III | Human | Italy | [37] |
|  | AY072728 | B | B IV | Human | Italy | [37] |
|  | AY545647 | D | D I | *Canis familiaris* | Italy | [38] |
|  | AY545648 | D | D II | *Canis familiaris* | Italy | [38] |
| *Giardia muris* | AF069565 |  |  | Mouse | USA | [42] |

**References:** 41. Trout JM, Santín M, Greiner E, Fayer R. Prevalence of *Giardia duodenalis* genotypes in pre-weaned dairy calves. Vet Parasitol. 2004;124:179-86. 42. Monis PT, Andrews RH, Mayrhofer G, Ey PL. Molecular systematics of the parasitic protozoan *Giardia intestinalis*. Mol Biol Evol. 1999;16:1135-44.
