# Supplementary material for: Prevalence of intestinal parasites and molecular characterization of Giardia intestinalis, Blastocystis spp. and Entamoeba histolytica in the village of Fortín Mbororé (Puerto Iguazú, Misiones, Argentina)
Source: Parasit Vectors. 2021 Oct 1;14:510. doi: 10.1186/s13071-021-04968-z (PMC8485468; doi:10.1186/s13071-021-04968-z)
Supplement: Supplementary file 3 — Additional file 3: Table S3. List of Giardia intestinalis-positive samples from participants of Fortín Mbororé, Puerto Iguazú, Misiones (Argentina) identified by microscopy and qPCR that were also identified at the assemblage level using the β-giardin gene. [file 13071_2021_4968_MOESM3_ESM.docx]

**Additional file 3: Table S3.** List of Giardia intestinalis positive sample from participants of Fortín Mbororé, Puerto Iguazú, Misiones (Argentina) identified by microscopy and qPCR that were also identified at the assemblage level using de β-giardin gene.

| Code Sample | Assemblage | Sub-assemblage | Reference Sequence |
| --- | --- | --- | --- |
| F005 | D | D II | AY545648 |
| F016 | A | AIII | AY072724 |
| F057 | A | AIII | AY072724 |
| F075 | B | BIV | AY072728 |
| F077 | A | AIII | AY072724 |
| F105 | A | AIII | AY072724 |
| F112 | A | AIII | AY072724 |
| F118 | A | AIII | AY072724 |
| F121 | B | BIV | AY072728 |
| F144 | A | AII | AY072723 |
| F145 | B | BIV | AY072728 |
| F158 | B | BIV | AY072728 |
| F163 | B | BIV | AY072728 |
| F174 | B | BIV | AY072728 |
| F179 | B | BIV | AY072728 |
| F181 | B | BIV | AY072728 |
| F183 | B | BIV | AY072728 |
| F193 | B | BIV | AY072728 |
| F195 | B | BIV | AY072728 |
| F196 | B | BIV | AY072728 |
| F199 | B | BIV | AY072728 |
| F214 | B | BIV | AY072728 |
| F255 | A | AIII | AY072724 |
| F277 | B | BIV | AY072728 |
| F280 | B | BIV | AY072728 |
| F282 | B | BIV | AY072728 |
